# Polysialic acid-functionalized liposomes for efficient honokiol delivery to inhibit breast cancer growth and metastasis

**DOI:** 10.1080/10717544.2023.2181746

**Published:** 2023-02-21

**Authors:** Xin Li, Shuang Guan, Henan Li, Dong Li, Dan Liu, Jing Wang, Wenquan Zhu, Guihua Xing, Liling Yue, Defu Cai, Qi Zhang

**Affiliations:** a Institute of Medicine and Drug Research, Qiqihar Medical University, Qiqihar, P.R. China; b College of Pharmacy, Qiqihar Medical University, Qiqihar, P.R. China; c College of Pathology, Qiqihar Medical University, Qiqihar, P.R. China

**Keywords:** Breast cancer, metastasis, honokiol, polysialic acid, liposomes

## Abstract

To improve the anti-metastasis effects of honokiol (HNK) on breast cancer, we designed cationic liposomes (Lip) in which HNK was encapsulated into Lip, and its surface was modified with negatively charged polysialic acid (PSA-Lip-HNK) for efficient treatment of breast cancer. PSA-Lip-HNK possessed a homogeneous spherical shape and high encapsulation efficiency. *In vitro* 4T1 cell experiments indicated that PSA-Lip-HNK increased cellular uptake and cytotoxicity via the endocytosis pathway mediated by PSA and selectin receptors. Furthermore, the significant antitumor metastasis impact of PSA-Lip-HNK was confirmed by wound healing and cell migration and invasion. Enhanced *in vivo* tumor accumulation of the PSA-Lip-HNK was observed in 4T1 tumor-bearing mice by living fluorescence imaging. For *in vivo* antitumor experiments using 4T1 tumor-bearing mice, PSA-Lip-HNK exhibited a higher tumor growth and metastasis inhibition compared with unmodified liposomes. Therefore, we believe that PSA-Lip-HNK well combined biocompatible PSA nano-delivery and chemotherapy, providing a promising drug delivery approach for metastatic breast cancer therapy.

## 1. Introduction

Tumor metastasis development is a multistage process in which malignant cells spread from tumor origin to colonize distant organs, which is the major contributor to most cancer patient mortality (Luo et al., 2020). Breast cancer is the most prevalent cancer among women (Siegel et al., 2023). The results of the American Cancer Society on breast cancer estimated that over 3.8 million women live in the United States with a history of invasive breast cancer, and approximately 270,000 women were newly diagnosed at the beginning of 2019. Over 150,000 breast cancer survivors have metastatic disease (Akram et al., 2017; DeSantis et al., 2019; Miller et al., 2019). Chemotherapy remains an essential approach to treating metastatic breast cancer, but its mortality rate is growing due to systemic toxicity and associated side effects (Qi Zhang, 2018). As a result, much attention has been paid to the discovery of new anticancer compounds.

Honokiol (HNK) is a small biphenyl lignan with biological activity extracted from the bark of *Magnolia officinalis* or other species of Magnoliaceae (Wu W & Xu, 2021). Applying HNK has demonstrated antiangiogenic, anti-inflammatory, antioxidative, antiviral, neurological effect, and antitumor activity in clinical studies, while safety and toxicology of HNK have revealed no detectable toxicity (Sarrica et al., 2018; Khatoon et al., 2022). Among these, its antitumor properties have garnered considerable research interest. HNK can inhibit tumor invasion and metastasis via modulation of EGFR, P53/AKT/PI3K/mTOR, and NF-κβ signaling pathways (Rauf et al., 2018; Li et al., 2020; Sayeh & Hassan, 2022). It also displayed efficient induction of apoptosis via triggering mitochondrial dysfunction and endoplasmic reticulum stress (Yeh et al., 2016; Shi et al., 2020). Some other research demonstrated that HNK could inhibit breast cancer cell migration by targeting nitric oxide and cyclooxygenase-2 (Singh & Katiyar, 2011). We previously discovered that HNK could inhibit cell migration and invasion in 4T1 cells by regulating E-cadherin and Vimentin expression (Zhang Q et al., 2020). However, due to the presence of phenolic hydroxyl groups in its structure, HNK exhibits poor water solubility and low bioavailability that restricts its clinical applicability for tumor treatment (Wang L et al., 2019). To address the problem of improving its solubility and bioavailability, it is critical to developing novel strategies.

So far, as nanotechnology has advanced, nanoformulations have become small in size and have a long circulation time (Yang et al., 2021). They are highly suitable vehicles for delivering anticancer drugs, significantly increasing drug accumulation in tumor sites (Wu P et al., 2019). Among these, liposomes were the most successful nanoformulation for clinical transformation. The drug encapsulated in liposomes can bring many benefits. Especially, it can increase drug solubility and improve its bioavailability (Magar et al., 2022). On the other hand, it can use liposome-cell membrane fusion property to deliver drugs into cells (Ju et al., 2018). However, the liposomes are easily captured by human reticuloendothelial system and cleared *in vivo*, reducing the drug’s efficacy. A key new strategy for resolving this issue is to modify ligands on liposomes to specifically recognize the receptors or epitopes overexpressed on targeted cells (Hatakeyama et al., 2011). Thus, the modification can increase the selectivity and uptake of liposomes in targeted cells, and prolong liposomes’ circulation time *in vivo* (Bae & Park, 2011; Allen & Cullis, 2013). Some small molecule ligands, peptides, and antibodies have been widely utilized in liposomes for their therapeutic effects improvement (Nel et al., 2023).

Polysialic acid (PSA) is an unusual linear polysaccharide, comprised of negatively-charged *N*-acetylneuraminic acid residues, linked specifically via α-2, 8- and α-2, 9-glycosidic bonds (Zhang T et al., 2016; Guo et al., 2019). As an endogenous and well-characterized carbohydrate polymer, PSA has the advantages of excellent hydrophilicity, non-immunogenicity, excellent biocompatibility and biodegradability, which is a recognized ideal ligand (Zhang Q et al., 2023). Furthermore, the monomeric unit of PSA (SA) can actively target tumor cells, serving as a targeting ligand for selectin receptors, which are highly expressed on tumor cells and tumor vascular endothelial cells. Thus, the feature of PSA is very beneficial for drug delivery. It could be used as a targeting ligand for the drug delivery system to improve the distribution of antitumor drugs in the tumor. It has been reported that PSA modified nanomedicines showed great potential in prolonging circulation half-life and reducing drug elimination rate *in vivo* (Wang XJ et al., 2019; Zhang Q et al. 2022).

**Scheme 1.**
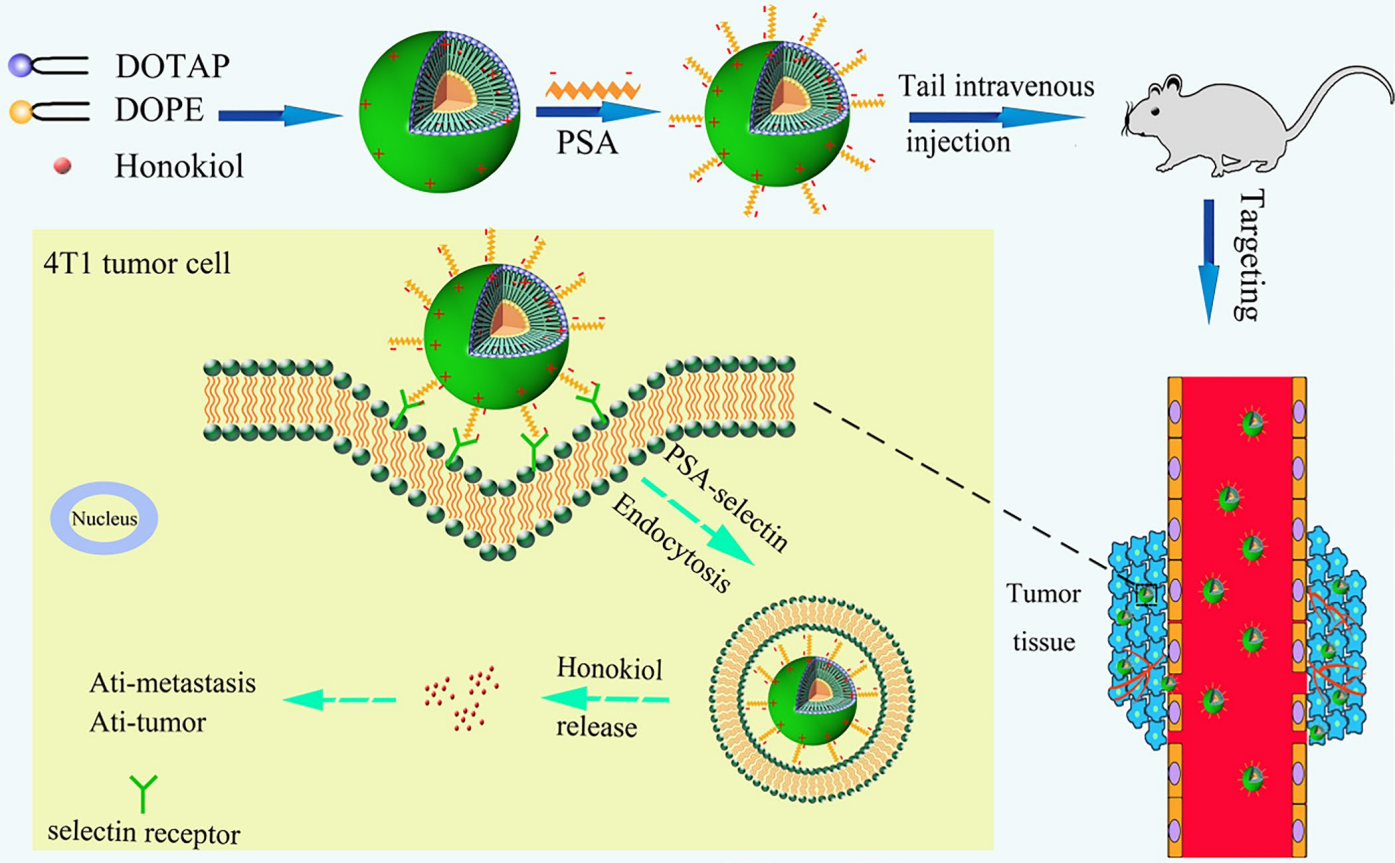
Scheme of *in vivo* tumor targeting and antitumor effect verification of HNK delivered by PSA-modified liposomes.

Considering these unique advantages of PSA, we hypothesized that PSA polysaccharides-modified liposomes could result in an improvement of the circulatory stability and preferential tumor tissue accumulation, providing a desirable curative effect for existing therapeutics. In this regard, cationic liposomes modified with PSA were formulated and loaded with HNK (PSA-Lip-HNK) aiming at improving its biodistribution and inhibiting tumor growth and metastasis (***Scheme 1***). And the size, morphology, encapsulation efficiency (EE), and drug release behavior of PSA-Lip-HNK were investigated. Subsequently, we examined the *in vitro* cytotoxicity and cellular uptake effects of PSA-Lip-HNK on 4T1 tumor cells. Tumor cell migration and invasion effects of PSA-Lip-HNK were also investigated. The antitumor activity of PSA-Lip-HNK was further confirmed in 4T1 tumor spheroids. Finally, a breast cancer model in mice was established, and the feasibility of PSA-Lip-HNK was validated in terms of *in vivo* biodistribution, antitumor, and antimetastasis efficacy.

## 2. Materials and methods

### 2.1. Materials

HNK was purchased from Dalian Meilun Biotech Co., Ltd. (Dalian, China). Egg yolk phosphatidylcholine (EPC) was provided by LIPOID GmbH Ludwigshafen, Germany. 1, 2-Dioleoyl-3-trimethylammonium-propane (chloride salt) (DOTAP) and dioleoyl phosphoethanolamine (DOPE) were supplied by Xi’an Ruixi Biological Technology Co., Ltd. (Xi’an, China). PSA (with MW of 80 kDa) were produced by Zhenjiang Changxing Pharmaceutical Co., Ltd. Cholesterol, Sephadex G-50, coumarin-6 (Cou6), Hoechst 33258, sulforhodamine B (SRB), tri-chloroacetic acid (TCA), and Tris base were all purchased from Sigma-Aldrich (Shanghai, China). The fluorometric TUNEL apoptosis detection kit was purchased from Promega (Wisconsin, USA). RPMI-1640 medium and penicillin–streptomycin were obtained from Macgene (Beijing, China). Fetal bovine serum (FBS) was purchased from Wisent (Nanjing, China).

### 2.2. Preparation and characterization of drug-loaded liposomes

The drug-loaded liposomes were prepared through the thin-film hydration method. Briefly, the EPC, DOPE, DOTAP, and cholesterol at a molar ratio of 2:1:1:1 were dissolved in dichloromethane to form a clear solution. Then the solvent was removed by vacuum evaporation using a rotary evaporator at 37 °C for 1 h. PBS (pH 7.4) was added into the mixture of lipid film, followed by sonication with the aid of an ultrasonic cell pulverizer at 50 °C for 10 min. The final Lip-HNK was obtained by filtering through a 0.22-μm filter to remove the unencapsulated HNK. The PSA-functionalized liposomes were prepared by rapidly adding liposomes into 0.6 mg/mL PSA solution (1:1, vol:vol) under stirring at 37 °C. Cou6 and DiR-labeled liposomes were prepared in the same way as HNK-loaded liposomes, excepting that HNK was added instead of Cou6 and DiR. The mean diameter and ζ potential of liposomes were measured using the dynamic light scattering analysis (Nicomp 380 ZLS, PSS, USA). The morphology of liposomes was characterized by transmission electron microscope (TEM). The EE of HNK in liposomes was determined using an HPLC system (Waters, USA).

To evaluate the rate and profile of HNK release from PSA-functionalized liposomes (PSA-Lip-HNK), *in vitro* drug release assay was performed using a dialysis method. Briefly, 1.0 mL of Lip-HNK and PSA-Lip-HNK solutions were put into a dialysis bag (10-12 kDa cut-off) having 50 mL release media composed of 100 mM PBS (pH 7.4) and 0.5% Tween 80 stirred at 37 °C for 48 h. At different time points up to 48 h, 1 mL of the dialyzing medium was withdrawn and replenished with the same volume of fresh release media. The concentration of HNK released was determined using the developed HPLC method.

### 2.3. 4T1 cell culture and animals

4T1 (murine mammary carcinoma cells, NICR, China) were cultured in RPMI-1640 medium (Macgene, China) supplemented with 10% FBS (Wisent, China) and penicillin–streptomycin (100 IU/mL and 100 μg/mL, Macgene, China) and kept in an incubator at 37 °C and 5% CO_2_. Female BALB/c mice of 18–20 g were obtained from Liaoning Experimental Animal Resource Center (Benxi, China), and housed at 20–25 °C and 50%–60% relative humidity with free access to food and water.

### 2.4. Cellular uptake and targeting effects

Flow cytometry was employed to determine cell uptake following Cou6 encapsulation into liposomes. 4T1 breast cancer cells were inoculated into a 6-well plate (1 × 10^5^ cells/well) and were cultured for 24 h at 37 °C. A serum-free medium containing Cou6 related preparations (free Cou6, Lip-Cou6, and PSA-Lip-Cou6) was added at a final Cou6 concentration of 100 ng/mL, and cells with formulations were then incubated for 1 h. 4T1 cells were washed with cold PBS three times, digested with trypsin, terminated with medium containing serum, and finally collected using 0.4 mL PBS. Cou6 quantitative analysis was performed using flow cytometry. Briefly, 1 × 10^4^ cells were collected and detected with an excitation wavelength of 488 nm and a detection wavelength of 560 nm.

**Figure 1.**
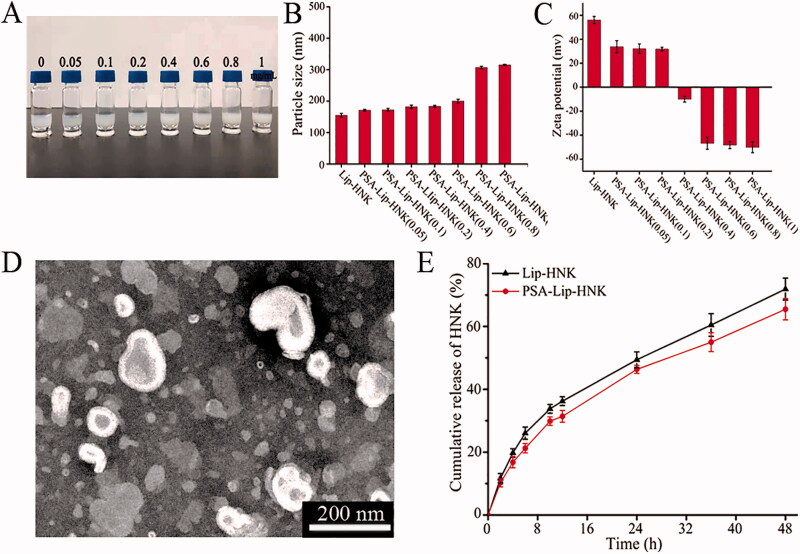
The characterization of the PSA-Lip-HNK. A: The appearance of PSA-Lip-HNK at different PSA modified density. B and C: The impact of PSA density on particle size and zeta potential of liposomes. D: Typical TEM image of PSA-Lip-HNK. E: *In vitro* release profiles of Lip-HNK and PSA-Lip-HNK in PBS at 37 °C. Data are presented as mean ± SD (*n* = 3).

We next employed laser confocal microscopy to perform qualitative analysis on the cells. The cells were treated with various Cou6 formulations and incubated at 37 °C for 1 h. The cells were washed with cold PBS, fixed with 4% paraformaldehyde, and then stained with Hoechst 33258 dye. Finally, the fluorescence signals in the cells were analyzed using a confocal microscope.

To verify whether PSA-Lip-Cou6 was specifically taken up by the 4T1 cells through receptor-mediated endocytosis, we designed a receptor competitive experiment in which excessive free PSA was added to block selectin receptors on the cell surface before incubation with PSA-Lip-Cou6. Specifically, 4T1 cells were preincubated with free PSA (1 mg/mL) for 1 h before the addition of PSA-Lip-Cou6. All other experimental conditions were the same as the flow cytometry and confocal microscopy analyses mentioned earlier. This treatment group was denoted as PSA + PSA-Lip-Cou6.

### 2.5. *In vitro* cytotoxicity studies

SRB method was used to investigate the cytotoxic effects of Lip-HNK, PSA-Lip-HNK, and free HNK on 4T1 cells. Specifically, 4T1 cells were cultured at 3 × 10^3^ cells/well in a 96-well plate and incubated in a humidified atmosphere of 5% CO_2_ at 37 °C. Following that, different HNK formulations were added to the wells at varying concentrations. After 48 h, the cells were fixed with 10% cold TCA and stained with 0.4% SRB. Finally, 10 mM Tris base was added to dissolve SRB dye. The optical densities were measured using a plate reader at 540 nm.

### 2.6. Cell migration and invasion assay

In the scratch wound healing experiment, 4T1 breast cancer cells were plated into a 6-well plate and scratched with a pipette after 24 h of incubation at 37 °C. After that, various formulations containing HNK (5.0 μg/mL) were added into corresponding wells. Finally, the wounded areas were imaged using an inverted fluorescence microscope at 0, 12, and 24 h after treatment.

To further observe cell migration, various HNK (5.0 μg/mL) formulations were incubated with 4T1 cells for Transwell migration and invasion assays. Typically, 100 μL cell suspension was added to the top of the Transwell chamber at a density of 5 × 10^4^ cells/chamber. Simultaneously, 600 μL complete RPMI-1640 medium was added to the lower chamber and incubated for 24 h. After rinsing the chamber with PBS, the remaining cells in the top chamber were removed using a cotton swab. The cells were fixed with 4% paraformaldehyde for 30 min and then stained with 0.5% crystal violet for another 30 min. The Transwell membrane was observed under an inverted microscope. Finally, 10% acetic acid was used to dissolve crystal violet on the stained cells, and absorbance was measured at 570 nm.

In the experiment of cell invasion, 8 × 10^4^ pretreated cells were added to the upper layer of the Matrigel-coated Transwell chamber and incubated for 48 h. The subsequent processing steps were the same as mentioned in the migration assay.

### 2.7. Growth inhibition effect on three-dimensional (3D) tumor spheroid

The hanging drop method was used to construct 4T1 cell tumor spheroids. The bottom of 48-well plate was precoated with 2% agarose, and 0.9 mL complete RPMI-1640 medium was added and left overnight at room temperature. Following that, 1 × 10^3^ 4T1 cells (20 μL) were suspended on the lid of 48-well plate for 48 h. The formed tumor spheroids were transferred into the hole and cultured for approximately three days. The tumor spheroids were treated with free HNK, Lip-HNK, and PSA-Lip-HNK at a concentration of 10 μg/mL HNK. The major (*d*_max_) and minor (*d*_min_) diameters of each tumor spheroid were measured, and the volume was calculated.

### 2.8. *In vivo* studies on biodistribution using fluorescence imaging

Female BALB/c mice (6–8 weeks, 18–20 g) were subcutaneously inoculated with 100 µL of 4T1 cells (1 × 10^6^ cells) in the right flank of mice to establish a 4T1 tumor-bearing mouse model. When tumor reached an average volume of 400–500 mm^3^, mice were injected with Lip-DiR and PSA-Lip-DiR through the tail vein. Fluorescence scanning was performed at 2, 4, 6, 12, and 24 h following intravenous injection using an IVIS Lumina Series III *in vivo* imaging system. Subsequently, the mice were sacrificed, and major organs together with tumors were collected. The fluorescent signal of each organ was determined by the IVIS imaging system.

### 2.9. *In vivo* antitumor efficacy

The aforementioned method was employed to construct 4T1 tumor-bearing mice. When the tumor volume of mice inoculated with 4T1 cells was 100–150 mm^3^, the mice were randomly divided into four groups (6 mice each). Saline, free HNK, Lip-HNK, and PSA-Lip-HNK were injected into mice through the tail vein. All HNK formulations were tested at an equivalent dosage of HNK (15 mg/kg) every two days for a total of four times. At every two days interval, tumor volumes and body weights of mice were recorded. On day 15, the mice were dissected to isolate tumors and main organs for further assay. Lung tissues were photographed, and metastasis nodules in lung tissues were calculated to evaluate antimetastatic efficacy. The apoptosis level inside the tumor was further detected by fluorescence TUNEL staining assay kit and then observed on an inverted microscope.

### 2.10. Statistical analysis

All experiments were performed at least three times, and all values were presented as mean ± standard deviation (SD). Student’s *t* test was used to investigate the statistical significance. The differences were accepted as significant for *p*** **<** **.05, and very significant for *p*** **<** **.01.

## 3. Results and discussion

### 3.1. Preparation and characterization of drug-loaded liposomes

In this study, the cationic liposomes with positive charge were prepared to encapsulate the HNK, whereas PSA is a kind of polyanionic linear polysaccharide with negative charge. Therefore, the electrostatic adsorption between polyanionic PSA and the surface of cationic liposomes was the basis for the PSA-Lip-HNK preparation. As depicted in ***Figure 1***(A,B), we studied PSA concentration’s impact on particle size and stability of liposomes. The unmodified liposomes had a mean diameter of around 160.6 ± 0.3 nm, with a slightly turbid appearance. When PSA concentration was increased between 0.05 and 0.6 mg/mL, the particle size progressively increased. However, the particle size increased rapidly to over 300 nm when the PSA concentration was further increased to 0.8 mg and higher. Nanoparticles intended for long circulation should have a perfect particle size between 10 and 200 nm (Fang et al., 2006). Considering controlling the ideal particle size of nanoparticles and providing sufficient PSA targeting ligands, we used a PSA of 0.6 mg/mL as the optimal concentration. At this concentration, PSA-Lip-HNK had a particle size of 184.2 ± 2.1 nm with a narrow PDI of 0.218 ± 0.040, there was no presence of large aggregates. As demonstrated in Figure 1(C), Lip-HNK had a zeta potential of +33.13 mV, which gradually decreased as PSA concentration increased but decreased to –50.15 mV when PSA concentration reached 1.0 mg/mL. This potential reversal change of liposomes provided adequate repelling force among liposomes to form a stable system, thus avoiding the aggregation and deposition of the liposomes in the vessel wall (Liu D et al., 2021). It was widely known that zeta potentials with absolute values > 30 mV allowed for electrostatic stabilization (Bader et al., 2011). In summary, a significant increase in particle size and surface charge reversal confirmed that anionic PSA successfully coated the liposomal surface.

The morphology of optimized PSA-Lip-HNK was further evaluated by TEM. All particles shown in Figure 1(D) were determined to be spherical morphology, uniformly distributed particle size, and evident bilayer membranes. And their sizes were consistent with those obtained by dynamic light scattering analysis. All liposome formulations revealed high EE for HNK, ranging from 83.0% to 90.0%. The dialysis method was used to determine the release of HNK from Lip-HNK or PSA-Lip-HNK, and the release curves are presented in Figure 1(E). As the curves display, Lip-HNK and PSA-Lip-HNK exhibited a slow, sustained release process. Both liposomes indicated a biphasic pattern of HNK release, with a fairly rapid initial release followed by a slow release. HNK release from liposomes exhibited a relatively fast release phase, releasing 36.17% of HNK during the first 12 h, whereas the cumulative release value for PSA-Lip-HNK was 31.63%. At 48 h, the cumulative HNK release percentage reached 71.90% and 65.69% for Lip-HNK and PSA-Lip-HNK, respectively. These findings indicated that PSA existence slightly slowed down the release rate of HNK owing to the decreased lipid bilayer fluidity and membrane permeability.

### 3.2. Cellular uptake and targeting effects in 4T1 cells

**Figure 2.**
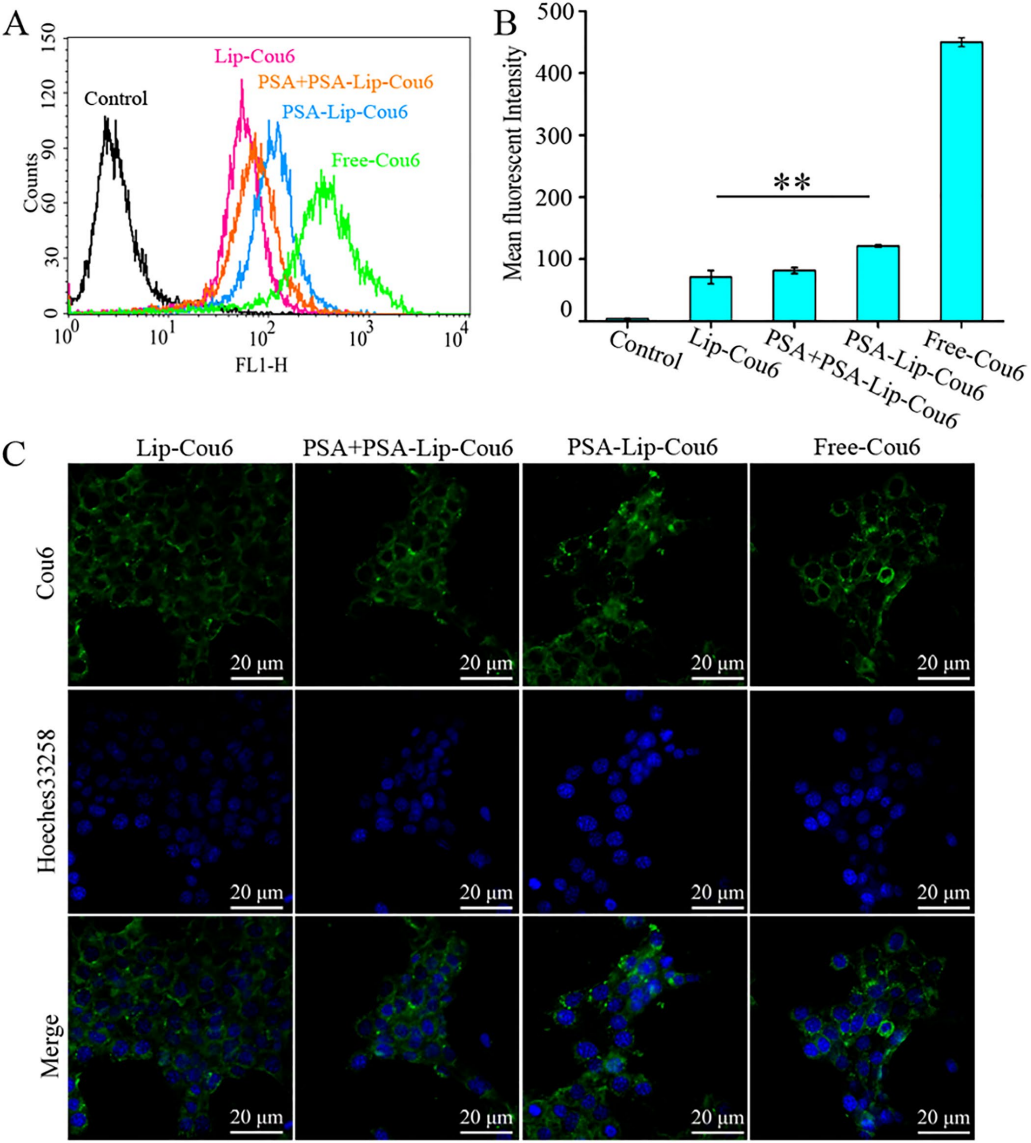
Intracellular uptake study of Lip-Cou6, PSA + PSA-Lip-Cou6, PSA-Lip-Cou6, and free Cou6 on 4T1 cells after incubation for 1 h at 37 °C. (A) Flow cytometric quantitative determination of Cou6 uptake. B: Quantitative analysis of Cou6 uptake based on flow cytometric plots. Each bar represents the mean ± SD (*n* = 3), **p < .01. C: Laser confocal scanning microscopy images of 4T1 cells. Green and blue indicate the fluorescence of Cou6 and Hoechst 33258, respectively.

Flow cytometry and laser confocal scanning microscopy were employed to evaluate *in vitro* cellular uptake of targeted liposome formulations in 4T1 cells. ***Figure 2***(A,B) demonstrated that the fluorescence signal of Cou6 in PSA-Lip-Cou6 group was stronger than in Lip-Cou6 group, the fluorescence intensity of PSA-Lip-Cou6 was 2.1 folds of Lip-Cou6. Since the composition was similar to that of Lip-Cou6 and PSA-Lip-Cou6 except for PSA, PSA modification was the reason for their obvious difference in cellular uptake. It has been assumed that the increased cellular uptake was probably caused by the PSA–selectin receptor-mediated endocytosis, hence a receptor competitive experiment was designed to provide more evidence. As shown in Figure 2(A,B), the enhanced fluorescent intensity by PSA modification was significantly reversed by the free PSA, indicating that specific binding of PSA and selectin would improve cellular endocytosis efficiency of liposomes. These results demonstrated that 4T1 cell endocytosis of PSA-Lip-Cou6 was greatly facilitated by PSA- and selectin-mediated internalization.

In addition, confocal microscopy images were utilized to evaluate PSA-Lip targeting ability. As illustrated in Figure 2(C), compared to Lip-Cou6, PSA-Lip-Cou6 treated 4T1 cells manifested stronger intracellular fluorescence intensity. In the competitive inhibition test, excess free PSA (PSA + PSA-Lip-Cou6) reversed the fluorescence intensity in cells. The aforementioned results indicated that PSA modification enabled Lip-HNK to specifically target 4T1 cells through selectin receptors.

### 3.3. *In vitro* cytotoxicity studies

**Figure 3.**
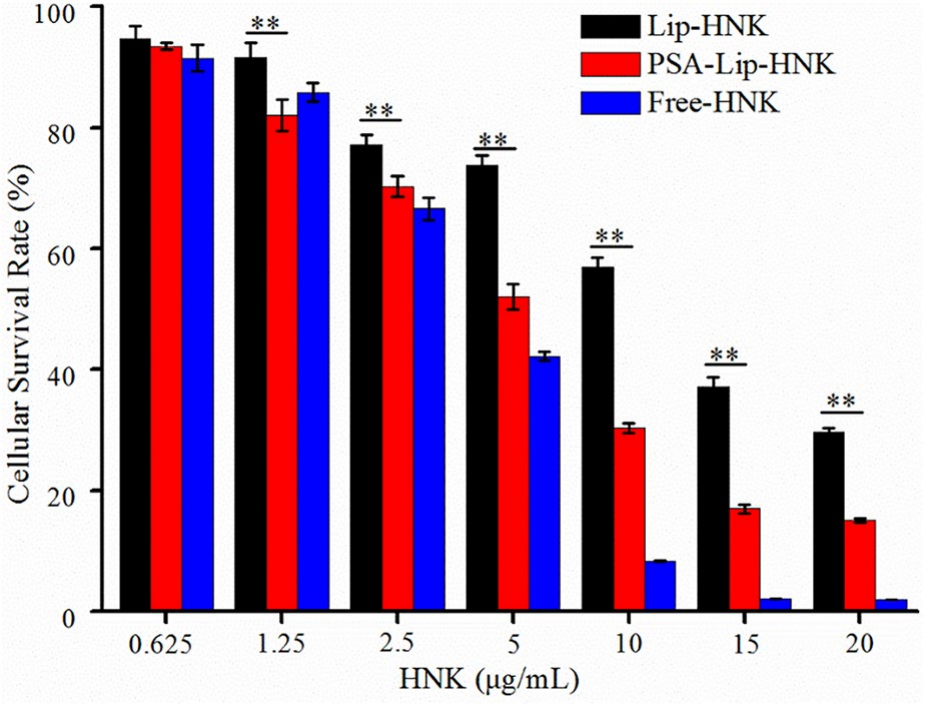
*In vitro* cytotoxicity against 4T1 cells of various HNK formulations after 48-h incubation by SRB assay. Each bar represents mean ± SD (*n* = 5). ** indicate statistically different *p* < .01.

The SRB assay was used to determine the cytotoxicity of Lip-HNK, PSA-Lip-HNK, and free HNK. ***Figure 3*** displays the cellular survival rates of 4T1 cells at a series of HNK concentrations. Obviously, free HNK, as a positive control, exhibited stronger cytotoxicity than Lip-HNK and PSA-Lip-HNK. Of note, PSA-Lip-HNK demonstrated stronger cytotoxicity on 4T1 cells than Lip-HNK, especially HNK concentrations in the range of 1.25–20 μg/mL. The results indicated that IC50 values of Lip-HNK, PSA-Lip-HNK, and free HNK on 4T1 cells were calculated to be 10.27 ± 0.29 µg/mL, 4.84 ± 0.18 µg/mL, and 3.29 ± 0.04 µg/mL, respectively. The IC50 value of Lip-HNK was 2.1 times higher than PSA-Lip-HNK. This implies that merely a low dose of PSA-Lip-HNK can achieve the same antitumor effect as a high dose of Lip-HNK. Altogether, these results suggested that selectin-mediated active targeting delivery using PSA-coated liposomes can reduce the HNK dose needed to achieve anticancer activity on 4T1 cells.

### 3.4. Evaluation of cell migration and invasion

**Figure 4.**
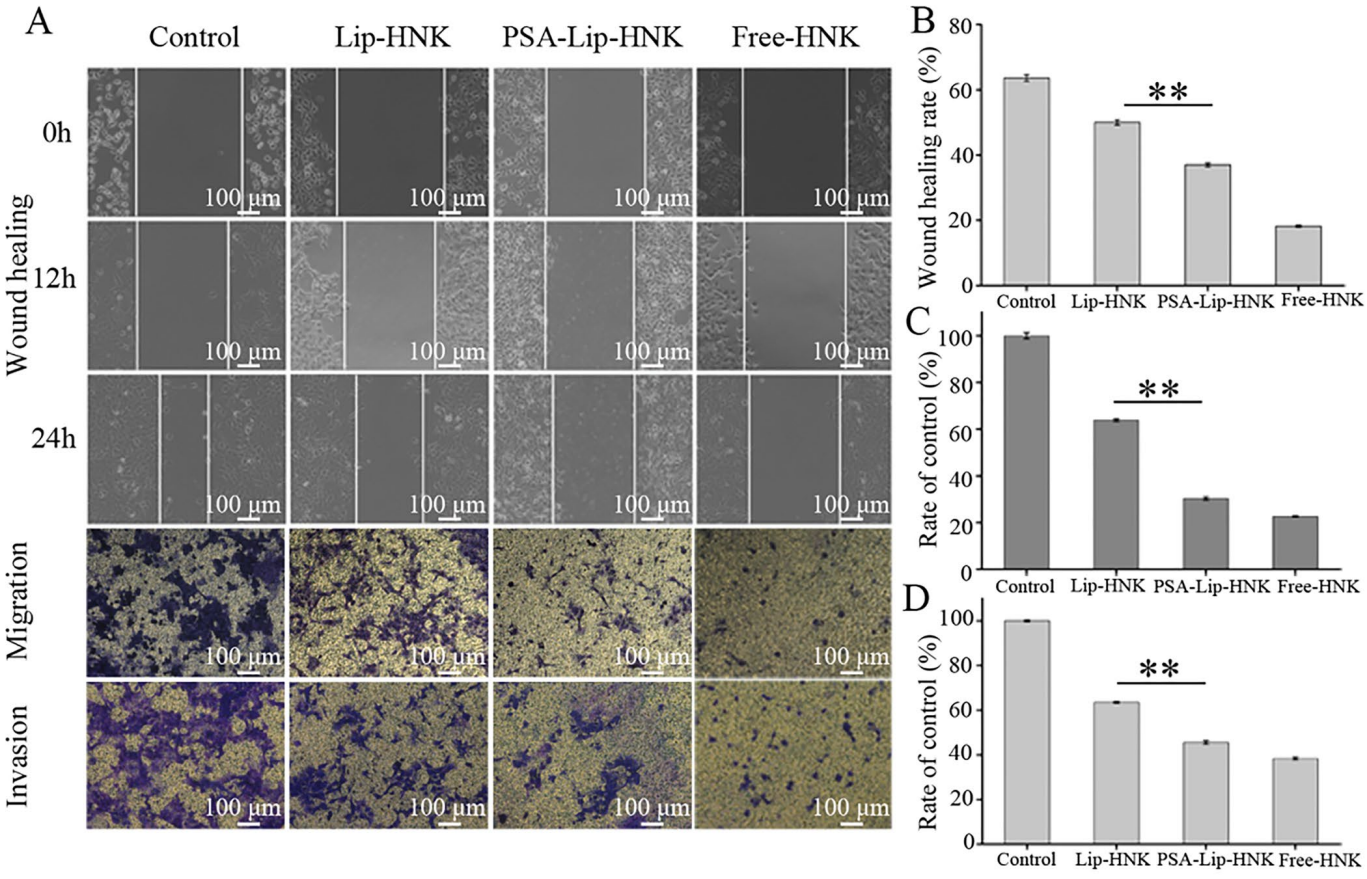
*In vitro* inhibitory effects of PSA-Lip-HNK on cell migration and invasion of 4T1 breast cancer cells. A: Typical images of wound healing, migration and invasion assessments of free HNK, Lip-HNK and PSA-Lip-HNK (5.0 µg/mL) on 4T1 cells. B–D: Quantified inhibitory effect of free HNK, Lip-HNK, and PSA-Lip-HNK (5.0 µg/mL) on 4T1 cells in comparison with negative control. Data are shown as mean ± SD (*n* = 3), **p < .01.

Like many cancers, metastasis is the leading cause of breast cancer death. Cancer cell migration and invasion are two key procedures of cancer metastasis (Mahauad-Fernandez et al., 2014; Yu et al., 2021). Therefore, to determine the ability of PSA-Lip-HNK in suppressing cancer cell migration and invasion, wound healing and Transwell assays were performed. As illustrated in ***Figure 4***(A,B), cells of the negative control group formed wound scratch healed rate of 63.7 ± 1.0% after 24 h, indicating a high metastatic potential in 4T1 cells. The free HNK as positive control exhibited the highest metastatic inhibition effect with wound healing rate of 18.0 ± 0.3%. The wound healing rates for Lip-HNK and PSA-Lip-HNK were 50.0 ± 0.6% and 37.2 ± 0.7% at 24 h, respectively. PSA-Lip-HNK exhibited a stronger capability for metastatic inhibition than Lip-HNK (*p*** **<** **.01).

Like in the wound healing results, similar trends were observed in the Transwell assay for PSA-Lip-HNK. Figure 4(A,C) shows that three HNK formulations treated groups had significant migration inhibitory effects compared to the negative control group. The migration ratio was about 30.3 ± 0.7% for PSA-Lip-HNK and approximately 63.8 ± 0.5% for Lip-HNK, and further reduced to 22.8 ± 0.1% in free HNK treated group. PSA-Lip-HNK produced a more obvious migration inhibitory effect than Lip-HNK. In the cell invasion experiment, Matrigel simulate the extracellular matrix of tumor tissue that was added on the upper chamber of Transwell (Qian et al., 2021). As can be seen in Figure 4(A,D), compared with the negative control, the invaded cells cross over the Matrigel-coated membrane was 63.5 ± 0.4% and 45.2 ± 0.9% in Lip-HNK and PSA-Lip-HNK treated group, and further significantly reduced to 38.4 ± 0.6% after the treatment of free HNK. These imply that the inhibition invasion ability of PSA-Lip-HNK was greatly enhanced compared to that of Lip-HNK. These results may be due to PSA moiety on the liposomes, which could produce more HNK uptake and make its potential as an excellent suppressor for tumor metastasis.

### 3.5. Growth inhibition effect on 3D tumor spheroid

**Figure 5.**
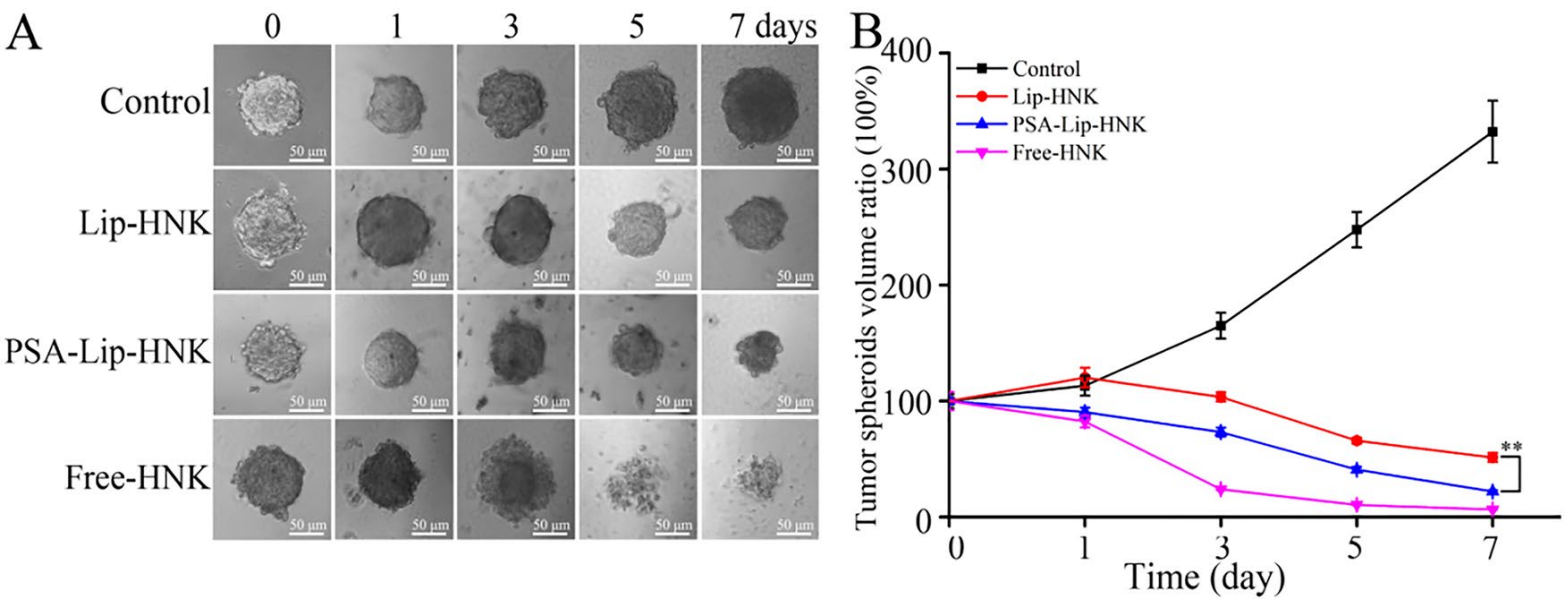
Effect of various HNK formulations (HNK = 10 μg/mL) on the growth of 4T1 tumor spheroids. A: Typical images of tumor spheroids under inverted microscope. B: The quantified size of tumor spheroids in different time points. Data are shown as mean ± SD (*n* = 3), **p < .01.

Unlike traditional monolayer cells, 3D tumor spheroids could simulate cell environment, cell–extracellular matrix, and cell–cell interaction, which are more likely to exhibit biochemical properties of tumors *in vivo*. Therefore, 3D tumor spheroids were increasingly recognized as a suitable tool to evaluate the efficacy of nanomedicine carriers for drug delivery (Perche & Torchilin, 2012; Kou et al., 2017; Liu et al., 2021). Representative optical images of 4T1 tumor spheroids treated with Lip-HNK, PSA-Lip-HNK, and free HNK at different time points are listed in ***Figure 5***(A,B). In the control group, tumor spheroids’ volumes ratio was 332.2 ± 26.8% on the 7th day, implying that tumor spheroids grow uncontrollably. As a result of diffusion effect, free HNK as positive control showed the strongest growth inhibition effect on tumor spheroids. Lip-HNK could reduce the volume to 51.3 ± 3.9% at day 7, which was markedly lower than in control group. PSA-Lip-HNK could be more effective in reducing the volume of tumor spheroids, which was 21.9 ± 1.1% on day 7 after treatment. These findings demonstrated that different HNK formulations could suppress tumor spheroids growth, while PSA-Lip-HNK had more potency than Lip-HNK. The excellent tumor spheroids growth suppression of PSA-Lip-HNK was supposed to be related to the PSA modification, which improved the penetration and accumulation of liposomes to tumor spheroids.

### 3.6. *In vivo* studies on biodistribution using fluorescence imaging

**Figure 6.**
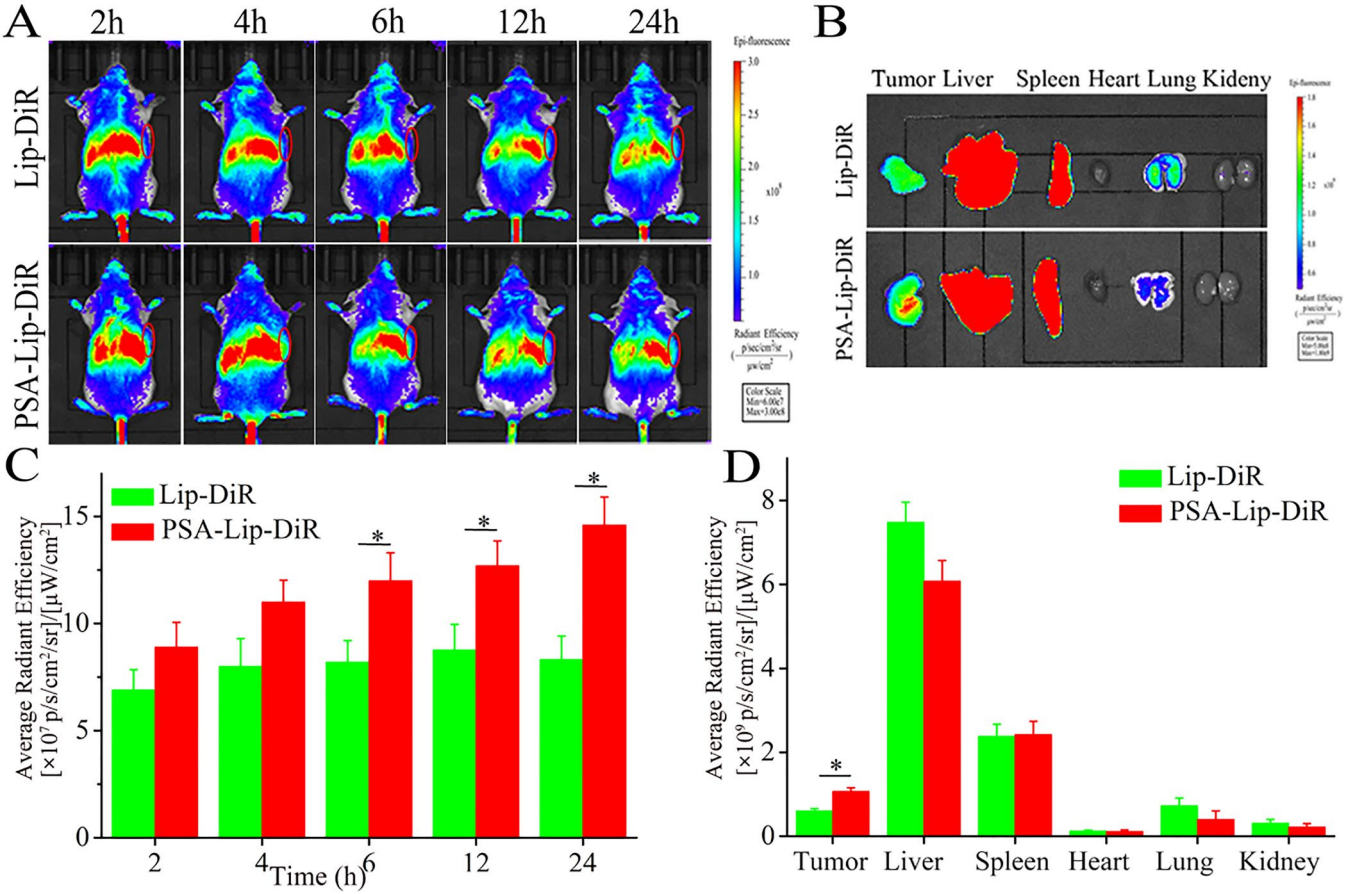
Biodistribution of the Lip-DiR and PSA-Lip-DiR in 4T1 tumor-bearing mice. A: *In vivo* real-time imaging of 4T1 tumor-bearing mice over 24 h after tail vein injection. B: *Ex vivo* fluorescence images of isolated tumors and organs. C: Semi-quantitative analysis of fluorescence intensity in the tumor site at the indicated time points. D: Semi-quantitative analysis of the *ex vivo* fluorescent intensity at the tumors and organs. Data are shown as mean ± SD (*n* = 3), *p < .05. The tumors were indicated by red ellipses.

The *in vivo* biodistribution of PSA-Lip-DiR in 4T1 tumor-bearing mice was monitored using living fluorescence imaging. According to the typical images shown in ***Figure 6***(A), PSA-Lip-DiR had a higher tumor retention and accumulation than Lip-DiR, owing to the PSA modification and PSA-selectin receptor mediated cellular internalization in the tumor. The strong fluorescent signals in PSA-Lip-DiR lasted until 24 h. In contrast, for the Lip-DiR, a marked decrease of fluorescence intensity in the tumor area was observed from 12 h after injection. At 24 h, the major organs and tumors of Lip-DiR and PSA-Lip-DiR treatment groups were isolated for further *ex vivo* image analysis. As shown in Figure 6(B), Lip-DiR and PSA-Lip-DiR exhibited strong fluorescence in the liver and spleen, indicating that a portion of liposomes was quickly captured by phagocytic cells of reticuloendothelial system (RES) in liver and spleen (Del Duca et al., 2004). However, PSA modification led to a reduced accumulation of liposomes in the lung, and an enhanced tumor accumulation, consistent with the results obtained by *in vivo* imaging of mice. The intratumoral fluorescence intensity of PSA-Lip-DiR was 1.76-fold higher than that mouse treated with Lip-DiR. In general, compared to Lip-DiR, PSA-Lip-DiR treatment resulted in preferential tumor accumulation, indicating that PSA modification can exert their active targeting ability and improve liposomes accumulation at tumor sites.

### 3.7. *In vivo* antitumor efficacy

**Figure 7.**
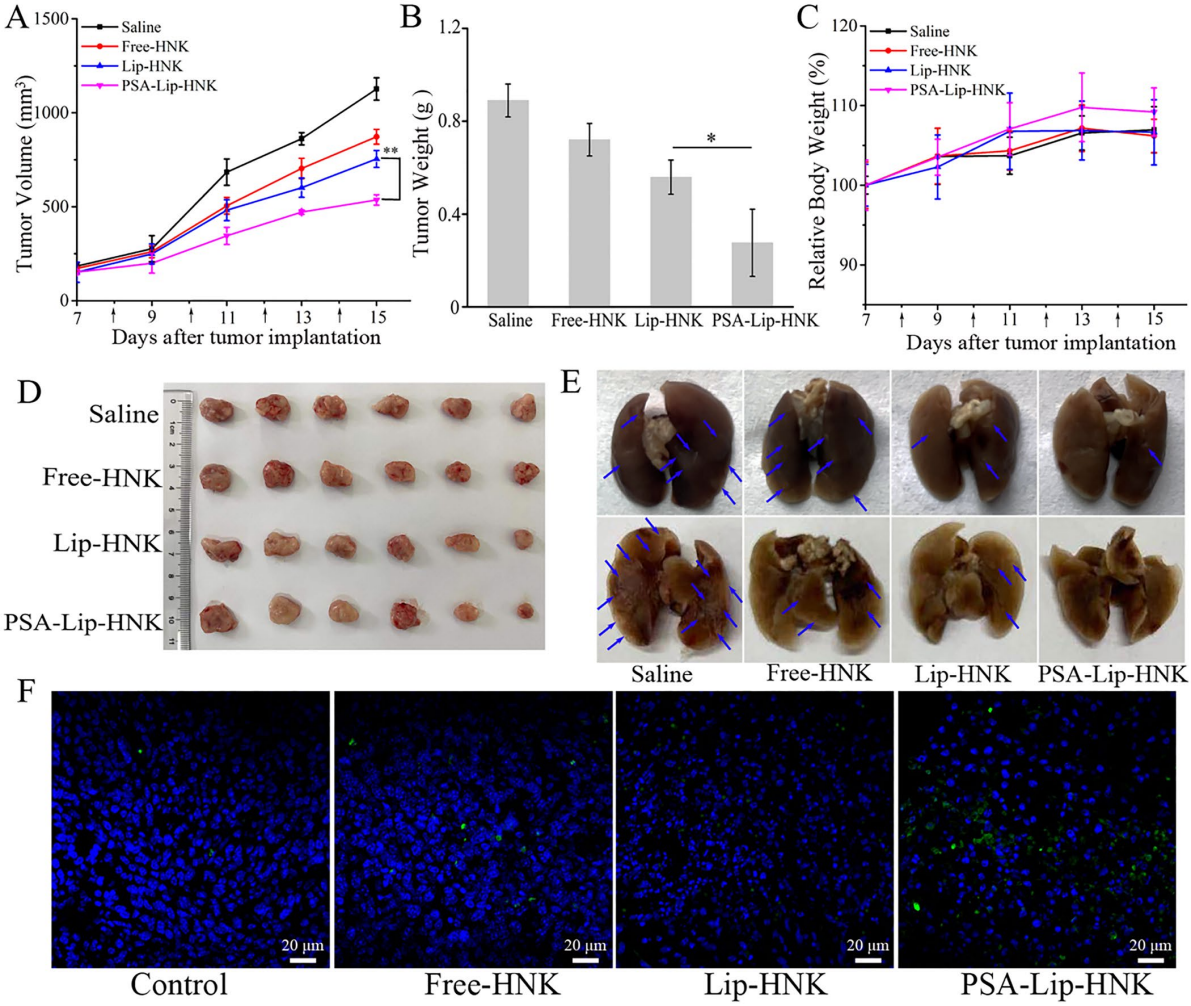
Antitumor efficacy of various HNK formulations (15 mg/kg) in 4T1 tumor-bearing mice models. A: Tumor volume change curves. B: The excised tumor weight. C: Body weight change curves. D: Images of excised tumors. E: Image of excised lungs (The pulmonary metastatic nodules are denoted by blue arrows). F: TUNEL fluorescent staining of apoptotic cells in excised tumor tissue. The various HNK formulations were injected every two days for the total of four times, data are given as mean ± SD (*n* = 6), *p < .05, **p < .01.

To evaluate *in vivo* antitumor efficacy of PSA-Lip-HNK, different HNK formulations were used to treat 4T1-induced metastatic breast cancer BALB/c mice models. As illustrated in ***Figure 7***(A), the tumor volume in the saline group increased sharply, while tumor volumes after three HNK formulations treatment were obviously smaller than that of saline. After four times of intravenous injections, the inhibitory ratios of tumor volumes were 22.56% for free HNK, 33.04% for Lip-HNK, and 52.40% for PSA-Lip-HNK. In particular, the PSA-Lip-HNK group exerted superior antitumor efficacy on tumor growth than Lip-HNK and free HNK groups. As demonstrated in Figure 7(B,D), PSA-Lip-HNK with the lowest tumor weight greatly inhibited tumor growth than other treatments, further verifying the superior antitumor efficacy of PSA-Lip-HNK. The superior antitumor efficacy of PSA-Lip-HNK is ascribed to two possible reasons: (1) nanosized liposomes promote HNK accumulation in tumor region by EPR effect; (2) the presence of PSA contributes to improve cellular uptake via PSA–selectin receptor mediated endocytosis.

**Figure 8.**
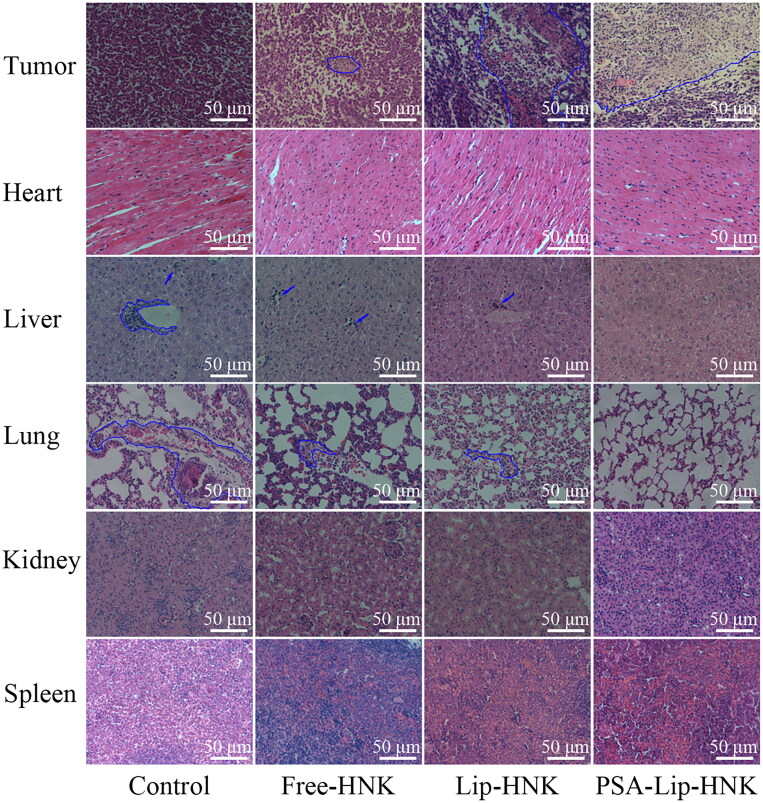
H&E staining photographs of tumors and main organs from different HNK treatment groups. The necrosis areas in tumor sections and metastasis lesions in liver and lung sections were circled by blue lines.

Moreover, fluorescence TUNEL labeling and H&E staining assays of tumor tissues were also performed to assess the antitumor efficacy of PSA-Lip-HNK. As shown in Figure 7(F), in comparison to saline treatment, a significant increase in TUNEL-positive signal (green) was observed in tumor sections treated with free HNK, Lip-HNK, and PSA-Lip-HNK. More importantly, the level of apoptosis after treatment by PSA-Lip-HNK was higher than the other two HNK groups, which was consistent with the results of tumor weight analysis. Furthermore, the result of the H&E staining assay was similar to that of the TUNEL. As shown in ***Figure 8***, remarkable karyolysis and cytoplasmic vacuolation were observed in tumor sections treated with three HNK formulations. Of note, the area of necrosis after treatment by PSA-Lip-HNK was largest than the other treatment groups, confirming that PSA-Lip-HNK promoted tumor cells apoptosis and suppressed tumor growth.

To ensure safety, the body-weight variation of mice and H&E staining were utilized as indicators of systemic toxicity. As depicted in Figure 7(C), compared to the saline group, no significant body weight loss was detected in various HNK formulation treatments, implying that PSA-Lip-HNK had no severe systemic toxicity. Furthermore, H&E histological examination (Figure 8) revealed that no pathological changes in the major organs of mice treated with PSA-Lip-HNK compared to those treated with PBS, indicating the biosafety of PSA-Lip-HNK. The aforementioned findings revealed that HNK and its PSA modified liposomal complex had good bio-compatibility and safety.

Metastasis usually occurs after advanced breast cancer, the lung is one of the most common metastatic locations (Autier et al., 2011; Kaushik et al., 2019). Therefore, encouraged by the superior tumor growth inhibition, the efficacy of PSA-Lip-HNK on breast cancer metastasis was investigated. As displayed in Figure 7(E), the saline treatment group mice developed extensive metastatic nodules in the lungs. Compared to the saline group, free-HNK and Lip-HNK treatments demonstrated a reduced number of pulmonary surface metastatic nodules. As expected, PSA-Lip-HNK treatment produced hardly visible metastasis nodule in lungs, implying the highest antimetastasis efficacy. In addition, the histopathological analysis of each organ section was performed using H&E staining to further validate the potential of PSA-Lip-HNK on antitumor metastasis (Figure 8). Similar to the trends of lung surface metastatic nodules, the lung and liver tissues of PSA-Lip-HNK treatment mice were looser than other treatments, and there was almost no tumor cell infiltration (with large nuclei) and dense deep staining variation. In summary, these results further verified that PSA-modified liposomal HNK could not only significantly inhibit the primary tumor growth, but also control distal tumor metastasis formation.

## 4. Conclusions

In the current work, PSA electrostatic modification of HNK-loaded liposomes was successfully fabricated and the resultant PSA-Lip-HNK was extensively characterized and tested in terms of cellular uptake, cytotoxicity, biodistribution, antitumor efficacy, and safety in different models. PSA modification enhanced the stability of the liposomal HNK and provided a better sustained release characteristic. Furthermore, PSA modified liposomal complex was better and faster internalized in breast cancer cells as a direct consequence of the PSA–selectin receptor mediated endocytosis. And this superior uptake resulted in enhanced cytotoxicity, anti-invasion, and anti-migration effects of PSA-Lip-HNK compared to Lip-HNK *in vitro. In vivo* biodistribution studies performed in the 4T1 tumor-bearing mice demonstrated that PSA-Lip-HNK resulted in preferential accumulation in the tumor site. Most importantly, the antitumor and antimetastasis activities of PSA-Lip-HNK were superior to that of the other formulations, which was likely attributable to that the presence of PSA endowed the liposomal HNK with a preferential tumor accumulation property. In addition, PSA-Lip-HNK had minimal systemic toxicity and no obvious tissue side effect. In summary, the developed PSA-modified liposomal HNK holds great promise for improving breast cancer therapy, which can be used to prevent primary tumor growth and control distal tumor metastasis formation.
